# Attention-deficit hyperactivity disorder medication shortage in the United States: a qualitative assessment of Reddit posts

**DOI:** 10.3389/fphar.2025.1529115

**Published:** 2025-07-07

**Authors:** Shikhar Shrestha, Shama Varghese, Simran Mehta, Saloni Dev

**Affiliations:** Department of Public Health and Community Medicine, Tufts University School of Medicine, Boston, MA, United States

**Keywords:** attention-deficit hyperactivity disorder, stimulant, medication shortage, Reddit, qualitative research, stress and coping theory

## Abstract

**Introduction:**

Approximately 6% of the US population had been diagnosed with attention-deficit hyperactivity disorder (ADHD) in 2023, and approximately 33% of the patients received stimulants to manage it. Recent ADHD medication shortages in the United States have caused substantial disruptions in access to these medications. The objective of our study was to investigate how individuals respond psychologically and behaviorally to ADHD medication shortage in the United States using public discussions on Reddit.

**Methods:**

We extracted Reddit threads from “r/ADHD” that consisted of the keyword “shortage.” After identifying relevant threads, we qualitatively analyzed 16 threads relevant to our study to reach thematic saturation. We used a hybrid inductive–deductive thematic analysis grounded in stress and coping theory to perform a qualitative analysis of the posts between August 2021 and February 2024.

**Results:**

Our findings show that redditors consuming ADHD medication experienced significant cognitive, emotional, and functional impacts due to medication shortage (primary appraisal). Redditors reported challenges with accessing medication due to complex medical systems, unsupportive healthcare providers, financial strain, and limited understanding of the causes of the shortage (secondary appraisal). Redditors also reported a range of pharmacological (change in dosage/medication, skipping dosage, saving medication, caffeine, and nicotine) and non-pharmacological (meditation and exercise) changes to behavior to manage the shortage (coping strategies). In addition, the Reddit community fostered support, positivity, and resource-sharing to navigate the shortage.

**Conclusion:**

Our study highlights the urgent need for healthcare policies that support individuals with ADHD amid shortages, emphasizing the role of transparent communication from pharmacies, healthcare providers, and regulatory bodies.

## Highlights

• We used Reddit posts to examine the impact of ADHD medication shortage.
• ADHD patients faced limited options and costly alternatives due to shortage.
• Medication shortage impacted the quality of life and daily functioning.
• Reddit users shared pharmacological and non-pharmacological coping strategies.
• The Reddit community fostered support, positivity, and resource-sharing.

## Introduction

Attention-deficit hyperactivity disorder (ADHD) is a complex neurobehavioral disorder characterized by inattentiveness, hyperactivity, and impulsivity (Biederman, 2005). According to the Centers for Disease Control and Prevention (CDC), approximately 9.8% of children aged 3–17 years (approximately 6.05 million) had been diagnosed with ADHD between 2016 and 2019 (Danielson et al., 2022). A recent report based on the National Center for Health Statistics (NCHS) Rapid Survey System (RSS) estimates that in 2023, approximately 6% of US adults received an ADHD diagnosis (Staley et al., 2024). The management of ADHD involves a combination of approaches, which include medications, behavioral therapy, educational support, and lifestyle changes (Catalá-López et al., 2017; De Crescenzo et al., 2017). ADHD medications aim to manage symptoms associated with it, such as difficulty focusing, impulsivity, and hyperactivity (Wilens, 2006). Common types of ADHD medications include stimulants (such as methylphenidate and amphetamine) (Castells et al., 2011; Punja et al., 2016; Van der Oord et al., 2008) and non-stimulants (such as atomoxetine and guanfacine) (Childress, 2016; Yu et al., 2023). ADHD medications work by affecting the levels of neurotransmitters in the brain, particularly dopamine and norepinephrine, either by increasing monoamine neurotransmitter levels by inhibition of vesicular monoamine transporter 2 (VMAT2) (e.g., amphetamines) (Martin and Le, 2024) or by inhibition of the presynaptic norepinephrine transporter (e.g., atomoxetine). Alternatives such as bupropion and risperidone are also prescribed when stimulants are not well-tolerated or in the presence of severe behavioral problems. Approximately 35% of individuals with ADHD diagnosis received stimulant medication in 2022–2023 (Staley et al., 2024). These medications improve the attention, focus, and impulse control in individuals with ADHD. While the exact mechanism varies depending on the type of the medication, the overall goal is to enhance cognitive and behavioral functions.

In a joint letter on 1 August 2023, the US Food and Drug Administration (FDA) and Drug Enforcement Administration (DEA) announced a shortage of ADHD medications (mixed amphetamine salts: shortage date first posted 10/12/2022, lisdexamfetamine: shortage date first posted 07/14/2023, and methylphenidate: shortage date first posted: 07/26/2023, all currently in shortage as of May 2025) (U.S. Food and Drug Administration, 2022) in the United States, raising concerns about the availability and accessibility of these crucial medications (U.S. Food and Drug Administration, 2023). Reasons highlighted for the shortage included manufacturing issues, supply chain disruptions, increased demand, and regulatory challenges, with the DEA noting that manufacturers did not reach maximal production, leading to a shortage of 1 billion dosages. After the announcements, the DEA changed quota regulations to reduce the amount of medications that the manufacturers needed to keep in the inventory (Drug Enforcement Administration, 2023). On 1 November 2023, additional changes were announced, which required manufacturers to submit production timelines, apply for quotas quarterly instead of yearly, and require monthly reporting (Drug Enforcement Administration, 2023). Although the changes were aimed at improving the production, the shortage of medications persists well into 2025, posing a serious risk to individuals with ADHD.

In response to medication shortages and scarcity, patients may resort to stockpiling, seeking medication from multiple sources, seeking alternatives, skipping doses, stopping the use, or buying from illicit sources (Ow et al., 2022). These actions not only jeopardize the effectiveness of treatment but also expose patients to potential health risks, especially when obtaining medications from unregulated sources. The illicit drug market, filled with potentially adulterated substances, also poses additional risks (Friedman and Shover, 2023). Therefore, it is crucial to understand how individuals with ADHD cope with medication shortages in order to develop supportive strategies and minimize potential harm. Although studies have extensively focused on how medication shortages are managed on a large scale through a complex mixture of interactions between markets, policymakers, and stakeholders (Shukar et al., 2021; van Oorschot et al., 2022), there is limited research on how individuals impacted by medication shortages manage their health.

Examining individual health behaviors in response to medication shortages can be challenging. Specific studies that are designed precisely to examine health behaviors at the time of shortage are difficult to develop and conduct in time. Therefore, the use of other passive measures of surveillance could be vital in examining the responses of large populations to unique shocks such as medication shortages. The use of social media platforms in medical and public healthcare research has increased significantly in the past decade (Golder et al., 2017; Ngai et al., 2015; Skaik and Inkpen, 2020). Social media platforms allow users to post their thoughts, experiences, advice, and warnings to the community; foster a discussion of solutions and in many instances guide people to resources that could potentially help them (Chen and Wang, 2021; Smailhodzic et al., 2016). In many platforms, this can be done anonymously, thereby protecting the identity of the poster and fostering discussion of themes that could be stigmatized (Berger et al., 2005; Eschliman et al., 2024; Highton-Williamson et al., 2015; Kepner et al., 2022). Reddit is a social media platform with a large user base (approximately 60 million users and over 1 billion posts as of 2023). It is particularly suitable for discussion of topics that may be stigmatized or require some level of anonymity, and in recent years, published studies have extensively used Reddit discussion forums to examine topics ranging from opioid use, stimulant use, harm reduction, and substance use treatment (Bouzoubaa et al., 2024; Eschliman et al., 2024; Kepner et al., 2022; Overbeek and Janke, 2018; Pandrekar et al., 2018; Spadaro et al., 2022).

When individuals face acute or ongoing disruption in access to critical resources or services, their response to such a disruption includes the replacement of the resources or service with displacement. However, when the resource or service is irreplaceable or no alternatives exist, various psychological and behavioral changes can occur, the understanding of which is necessary for developing public health and policy interventions. In the context of ADHD medication shortage, the use of a behavioral model grounded in psychological and behavioral theory can help understand how people perceive and cope with such a stressor. While several theories could be applied in this scenario, the stress and coping theory developed by Lazarus and Folkman is particularly relevant as it examines how individuals appraise and respond to stressful situations (Lazarus, 1984). As outlined by the stress and coping theory, when faced with a stressor, a sequence of evaluations are undertaken by an individual: a primary appraisal to assess whether a situation poses a threat or benefit, followed by a secondary appraisal of resources and coping options. This is followed by the adoption of problem-focused and emotion-focused coping strategies. This model is, therefore, well-suited for application in examining medication shortages and coping strategies used by ADHD patients.

The overall objective of our study is to leverage the Reddit ADHD forums to gain insights into the impact of ADHD medication shortages on patients. By analyzing user discussions using the stress and coping theory, we aim to understand coping mechanisms, behavioral changes, and potential risks associated with the scarcity of ADHD medications. This research is crucial for informing healthcare policies and interventions to support individuals affected by the shortage, thus ensuring their wellbeing and minimizing adverse outcomes.

## Methods

**FIGURE 1:**
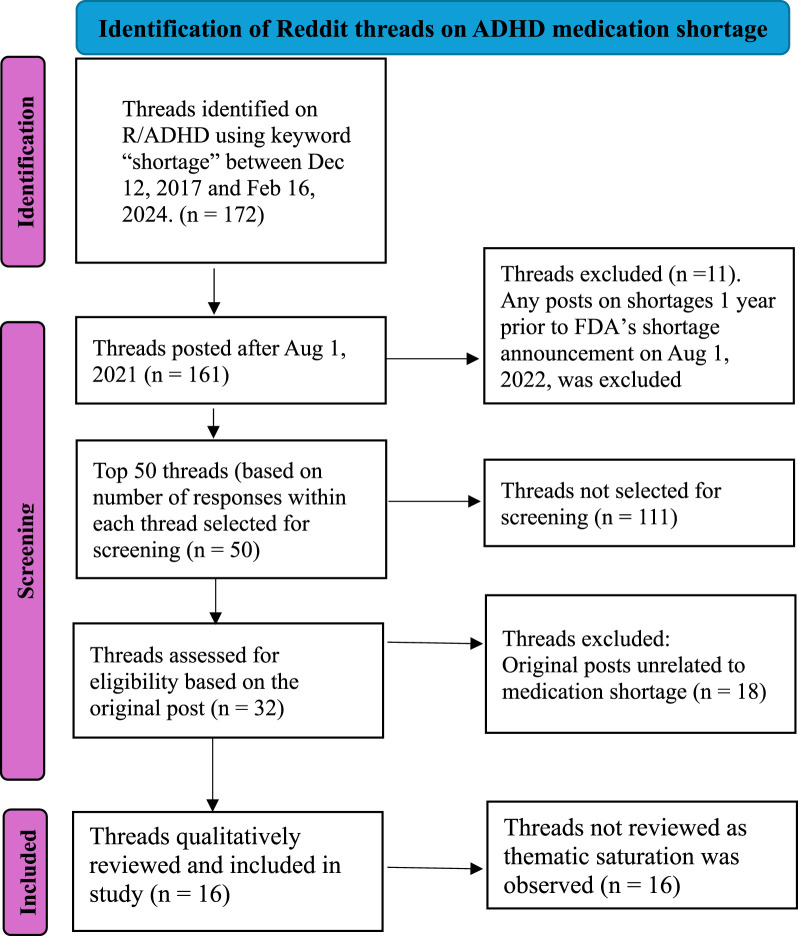
Flow diagram highlighting the identification and selection of Reddit threads on ADHD medication shortage included in the study.

Data: a subreddit is an online forum within Reddit that is dedicated to discussing a specific topic. The ADHD subreddit is a widely popular forum to discuss information on ADHD diagnosis, management, and treatment. We searched ADHD (“r/ADHD”) and collected the data of all publicly available posts that mentioned the term “shortage” using Python Reddit API wrapper (PRAW) on 16 Feb 2024. We identified 172 threads posted between 12 December 2017 and 16 Feb 2024. We excluded any original posts that were made before 1 August 2021. This start date was used to mark a 1-year period before the FDA’s announcement of the shortage. We identified a total of 161 threads based on our preliminary search criteria. The writing team reviewed the first post in the top 50 threads (sorted by the number of posts in a thread, with more posts indicating greater interaction and discussion) to establish the relevance of the post to medication shortage. We selected the top 50 threads as reviewing all 161 threads and replies within them would be too time-consuming and would have diminishing returns after a certain number of posts were reviewed. After excluding posts unrelated to ADHD medication shortage, we initially selected 32 threads and all posts within them for review, eventually reviewing and coding the 16 highest ranked threads for this study (Figure 1).

**FIGURE 2:**
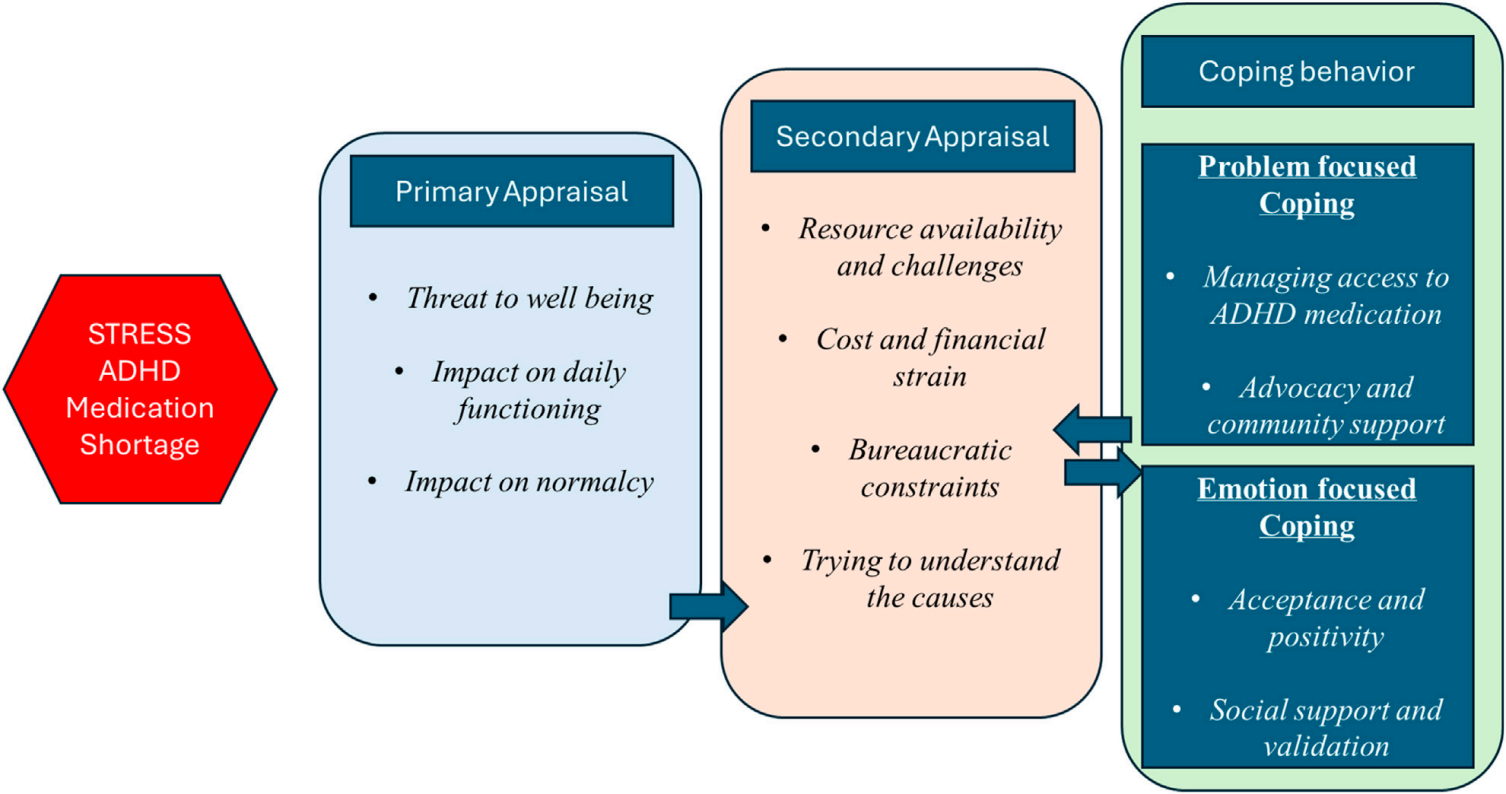
Operationalization of the impact of ADHD medication shortage using Lazarus and Folkman’s stress and coping theory.

Analytical framework: to understand the cognitive, behavioral, and social responses to ADHD medication shortage, we employed a hybrid inductive–deductive coding approach for a thematic analysis of the data. To generate *a priori* codes, we utilized the stress and coping theory. The stress and coping theory, developed by Lazarus and Folkman (Lazarus, 1984), focuses on how individuals perceive and respond to stressors, which could provide valuable insights into how people are coping with the challenges posed by the medication shortage. Upon encountering a stressor, individuals perform a primary appraisal, evaluating the stressors and determining if it could lead to a positive outcome, neutral outcome, or negative outcome or harm. After the primary appraisal, if the stressor is deemed to cause harm, then the secondary appraisal is carried out. Secondary appraisal includes evaluation of available resources and strategies to manage stress, evaluation of overall strain on health and finances, examination of the causes of stressors, and finding solutions to the stressor. The next step is coping strategies, which includes problem-based coping strategies and emotional coping strategies. Using the overall framework of the stress and coping theory, we developed initial codes for the thematic analysis (Figure 2).

Data extraction: first, analysis [SS] reviewed the highest-ranking thread “r/ADHD Megathread: Medication Shortages” and outlined the first set of codes using the stress and coping theory as the framework. The research team [SS, SM, SV, and SD] reviewed the initial codes and grouped them into the main categories based on the behavioral theory (primary appraisal identifying the impact of ADHD medication shortage on health and secondary appraisal explaining how people perceived the shortage, managed resources, or discussed the sources of shortage) and coping strategies such as behavioral changes, finding alternatives, and seeking social support. Any disagreement was noted and then addressed by the team.

To ensure that the coding framework was both theoretically informed and grounded in the data, the research team engaged in a collaborative immersion process. The research team reviewed 180 posts together to become familiar with the content of the posts, practice initial coding, and note new topics and ideas that emerged in the posts, in addition to the codes that originated from the stress and coping theory. The team later met to discuss the coding process and the new codes and drafted the final codebook. This process also ensured the comprehensiveness of the codebook and consensus among analysts. Following several rounds of discussion and refinement of the codebook, the remaining posts were distributed among two analysts (SS and SM) for individual coding until thematic saturation was achieved. Once thematic saturation was reached after reviewing 16 threads, our team examined the coded segments to identify patterns and recurrent themes, particularly focusing on medication shortage as a stressor, primary and secondary appraisals, and the coping strategies utilized. We did not perform inter-rater reliability assessments as each post within a thread was brief and included one or two key themes. Posts that were larger or included several themes were reviewed by the research team to highlight key themes.

## Results

Primary appraisal of the impact of ADHD medication shortage: redditors discussed the severe effects of ADHD medication shortage (Table 1). ADHD medications need to be taken daily to treat the condition, and patients are often on a unique regimen, deviation from which significantly impacts the quality of life. Many redditors reported not being able to fill their ADHD medication prescription for over a month. Redditors highlighted physical, emotional, and cognitive impacts leading to an increase in levels of stress and anxiety. Emotional impacts included an increase in stress, anxiety, depression, and suicidal ideation. Many redditors indicated experiencing “brain fog,” which affected their ability to think and complete tasks. This led to a significant impact on schoolwork, employment, and social relationships—many redditors stated that they could not cope at school and fell behind on coursework, and some reported having challenges at work that could potentially lead to their termination. Many redditors noted that the lack of ADHD medication significantly impacted their ability to look for ADHD medication, pulling them into an inescapable loop of despair.

**TABLE 1:** Primary appraisal of the impact of ADHD medication shortage.

Threat to wellbeing	Emotional impact	“So this is just going to keep going on forever then and we all just have to learn to live with it?? This has been an ongoing issue since October. Every single month causes so much anxiety and stress for me as my refill date approaches. I am so tired of this. This is beyond insane.” “…I take them as needed, usually every other couple of days, maybe every day on a hard week- and I’ve been having it out with life the past month or so- so I am now facing beginning stages of crisis because I cannot find it anywhere and on top of the stress it’s causing, it’s taking a toll on my focus too. I had no idea of the shortage and I wish I had seen this coming … ” “Also I get extra stressed when I take meds, and they do not click with my brain like normal, because I do not have enough to waste any, and that’s before considering trying to save any bc of the shortage. My refill is the day after my vacation ends, so I’m already dreading the stupid on hold music and 15 min/per phone calls to each of the walgreens in town to find where my adderall/any adderall is in stock. All that is before the stress of knowing that pharmacy might sell the pills I need while trying to get in contact with my psych to let her know what dose and location I need it sent to, then waiting so anxiously until she’s finished her business day and send them over. So I guess a lot of us are dealing with this stressful situation.”
	Physical impact	“I have a stash of about 2 weeks’s worth of my old, too low dose. I already am seeing a degradation of my attention, consistency, energy, emotional regulation, and also an uptick in all my bad habits to seek dopamine.” “…The shortage has put and end to my energy, my ability to motivate, my patients with her, and giving a shit about my own life. I made it through college without medication. But now my system is dependent on the drug to make dopamine. If I had it to do over, I would never have taken any drug that could be taken away from me at the whim of forces beyond my control. Think hard about that….”
	Cognitive impact	“I have a hard enough time focusing already, so I really cannot wait to also deal with the return of brain fog.” “My last refill was December 27th. My refill status is still considered “in progress” and has been since then (just says adderall is not in stock). ….My partner and I are trying to move out of his parents house and. I can barely get through the daily fog of my mind. It’s like my mind has reverted back completely to when I was in elementary school and barely cognizant of everything happening around me. It’s awful” “…Walgreens was able to fill, but my total amount with insurance is $288 for 30, 40 mg pills. That’s absurd! Anyway, my current status, have gone cold turkey, 3 days now. Day 1, I coped a little, still had Brain fog & no motivation. Day 2, slept basically the entire day. DAY 3 - feeling fog brained, fatigue, cannot focus nor concentrate while feeling anxious, sleepy & useless. I have a Dr. appointment in about 3 h to talk to my doctor about other possible alternatives. Can’t afford to pay $288 a month, … ”
Impact on daily functioning	Productivity/work	“This makes me want to just not even try to do more things in my life. Just when I was realizing my potential and trying I realize it could all come crashing down and I could loose my job and apartment because of lack of medication. It’s scary.” “I barely work 20 h a week and i still cannot function. Never took more than 5 mg of ritalin, but have not had access to it in months and my brain just does not [expletive] work.” “Has anyone gotten bad feedback at work or fired from their jobs? I got bad feedback and my boss wants to know what is going on ugh.”
	School	“My area has been out of stock for literally months and nobody has any estimate of when they might be able to get some in. My doctor switched me to Ritalin in the meantime, but it hardly does anything for me. I’m in grad school so I feel your pain, I’ve really been struggling.” “I started grad school in January, and have not been able to fill my concerta in 2 weeks now, im falling behind in class and am severely depressed. Why cannot i just push through and do it.” “Hi. I’m 15 and happier without my meds. However, without them I cannot FUNCTION IN SCHOOL. You know, the thing old people are always complaining we do not care about anymore? I do not take Ritalin to get high or whatever, I take it so that I can function as a person in this grind culture until I’m old enough to leave school. This just feels like adults making problems and blaming the recipients.”
	Relationships	“It’s incredibly not fun as an adult either, and I feel bad for my husband for having to deal with me while I’m not medicated.” “I’m in the same boat, the pharmacy was out of adderall, so I was perceived Ritalin, and I’ve called multiple pharmacy’s in my area, all gone. And these useless doctors will not call in for us to see if they have it so it just looks like I’m a drug addict seeking their next fix. As a result of this, my work life, social life, and relationship is suffering and idk when it’s gonna get better.”
Impact on normalcy	Challenges navigating day to day lives	“Three month wait for my scrip so far (this time around), and I’ve gained at least 30 pounds. I’ve also started smoking again, neglect my household duties far longer than I should, shop online for stuff I do not need, the list goes on and on. My impulsivity and anxiety are through the roof. My meds kept me from making stupid decisions, kept me organized, social, and overall allowed me to live a normal life.” “All I want is to go work and take care of myself and be a normal human and I’m not allowed. I cannot get my adhd meds. My shit county doctor refuses to prescribe me the benzos that I need and that allowed me to function and get over my crippling anxiety for 20 years. I’ve gone from having a place of my own and working regular jobs consistently to being homeless and occasionally being able to rent a room with crazy landlords and not being able to wok at all consistently.” “I have not been able to fill my Adderall prescription for 3 months. I keep forgetting to do even the most basic of chores to the point that my lovely, patient boyfriend has confessed that he’s exhausted from cleaning up after me. I’ve ruined my credit because of forgetting to pay my bills then getting so anxious about money that the thought of opening my banking app fills me with primal fear. I’m a financial burden on my boyfriend.”
	Challenges in getting ADHD medication because of the lack of ADHD medication	“And the irony of needing to call my doctor and a million pharmacies every month in the hopes of getting the medication that will enable me to call my doctor and a million pharmacies is … rich.” “This is my thought too. Any other disability, there would be hell breaking lose if medicine was not available. And trust me, there’s hell but we just do not have the motivation to make it break lose- BECAUSE WE DONT HAVE OUR MEDS!!!“ “Yeah I’ve only ever been prescribed Vyvanse and adderall IR. I just do not think the “it’s illegal to fill … ” supplemented with the “your dosage is too high” just makes it seem so off. I agree. He should of just called instead of what it seemed like badgering me.” “I just imagine the people who really need adhd meds to function properly and are needed to jump through hoops just to get their meds. Because let’s be real, us procrastinators definitely procrastinate before getting a script filled and before we know it we have no pills left.”

Secondary appraisal of the impacts of ADHD medication shortage: challenges in accessing ADHD medication, cost, bureaucratic constraints, and concerns about the source of the shortage were commonly reported by redditors (Table 2). The need to call multiple pharmacies to check for medication availability and then calling providers to transfer prescriptions, all when functioning without ADHD medication, which makes these performing these tasks difficult, was reported to be common. These were often insurmountable tasks, which were time-consuming and frustrating for many redditors. Some redditors reported no clear end in sight for the shortage. The lack of reliable information from pharmacies further complicated the situation, leaving individuals to call multiple pharmacies, hoping to find available medication. This was often compounded by the stigma faced by patients from the pharmacy and other healthcare providers as the patients had to call several pharmacies multiple times seeking refills for their ADHD medications.

**TABLE 2:** Secondary appraisal of the impact of ADHD medication shortage.

Resource availability and challenges	Calling multiple different pharmacies	“As someone who has been taking adderall for over a decade (minus my two pregnancies), I agree that it is super frustrating to see all these people who keep saying that they filled their rx immediately. In the entire time that I have been taking this, I have never had an issue filling it. I may have had to go to 2 pharmacies (like in Nov or Dec) before finding it, but nothing like this!! it is crazy to me! I called so many places today, and everyone told me they had nothing at all! I feel super defeated right now. Plus, I am not even really convinced that the people I talked to were even telling me the truth.” “This whole situation is messed up. The system is against us!!! Can’t refill your meds within 1–2 days before you run out cuz of rules - you’re lucky if the pharmacy has it, but most times not so they have to order it which can take days or weeks - oh you wanna call around to see if other pharmacy have it? Joke is on you! I’ve had so many pharmacists tell me they cannot disclose what they have in stock, but they do not care!!” “I called 13 different pharmacies today to find one that had it. Thank god I have not been taking my full 30 mg per day so I have a 30 days supply as backup.”
	Where medications are available and where medications are not available	I use a smaller local pharmacy. CVS and Walgreens have been out on and off for months, and with my CVS at least you get put on a list to get filled whenever they get it, first come first served. I waited 3 weeks the first time, but luckily I am on a twice a day so if I did not absolutely need the second one I did not take it, so it lasted a bit longer Definitely try smaller pharmacies! That’s what I had to do. Unfortunately in my town we do not have a wide variety to choose from, so I’ve kind of spread my family’s prescriptions out over a few different ones Try and check smaller privately owned pharmacies instead of CVS/Walgreens, etc. I’m in NJ and every CVS in the area has been out for months. The only place I’ve been able to fill it is this little pharmacy in a nearby town. I really feel for you and I would not be able to function without it either.
	Support from medical providers	“…my pharmacy did not have the 20 mL to filled mine. My doctor changed my prescription from 20 mL extended released to 10 mL take 2 in the morning.” “. ALL were out of Adderall. My doctor just switched me to Vyvanse for the time being during this shortage. I wish you the best during this trying time!” “I’ll be talking with my doctor on Thursday about getting on a non-stimulant that is not the ones I’ve tried previously (Clonidine and Buspar) because I do not want to add to the shortage but the way it’s going I do not think non-stims are going to help. My therapist and I do have a solid plan on how to move forward if I fail out of Strattera and Guanfacine though so hopefully it’ll be okay.”
	Stigma of using stimulants	“Something tells me addicts do not just keep forgetting to meet up with their drug dealers to get their fix. Some of these doctors really need to get their heads out of their [expletive].” “A lot of us cannot stock up. Since stimulants are so highly controlled, we’re only allowed to have our meds for the month at any given time. They think if they give us our meds ahead of time or give us extra meds we’ll sell them or something. [Expletive] that noise, that’s my MEDICINE, I’m not selling it to some grad student for a study drug. But here in the US they assume everyone is a degenerate or an addict or a drug dealer.” “I’m so f****** tired of EVERYONE thinking that if you express any interest in being on your proper medication. you’re obviously a drug dealer or addict! This crap has got to be addressed!!!! When I cannot be medicated legally through a physician but can have ANYTHING delivered illegally to my doorstep, the [expletive] are winning!” “My point being, many people turn to illicit substances to self-medicate. By taking away access and continuing to criminalize doctors who are quite literally *just doing their job*, while making their patients feel like criminals, they’re *making the goddamn substance use problem worse*. When people cannot access their meds legally, many will just turn back to what they did before.”
Cost and financial strain	Alternatives are expensive	“I switched insurance recently and something that was costing me $30 now costs me close to $400 I did not realize how expensive Vyvanse is I decided not to fill it.” “It’s either nothing for me, or 386$ out of pocket for Vyvanse. I got a huge promotion in November, and have not been able to fill my prescription since mid-November. I’m thankful for a great boss, but I waved my white flag and bought the Vyvanse. I feel like I have been a huge disappointment since my promotion. I worked towards this promotion for 3 years. My heart is hurting right now.” “It’s seriously awful. I went from having a $0 copay for Adderall XR to having to pay $300 a month for Dyanavel XR because it’s not on my formulary but nothing else really is that works SO I’m cash paying a 300% markup until who knows when. It’s insane.”
Bureaucratic constraints	Challenges transferring prescription	“The pharmacy cannot transfer scripts. The dr office has to transfer the script, because it’s a controlled substance” “Many states do not allow the transfer of prescriptions for controlled substances unfortunately. That’s why many people here are recommending trying to get a paper script so you can sneaker-net it around.” “If you want to try calling other pharmacies (if it’s a chain like CVS or Walgreens, you can ask if your pharmacist can check other locations, but it’s a complete crapshoot whether they’ll do it or not) and find one that has your meds in stock, you have to contact your doctor to ask them to transfer the prescription to the new pharmacy AND cancel the original prescription.” “I was able to find a pharmacist who actually checked their supply and magically they had a half dose that would cover me. You’d expect that they could easily swap it since they should be the same dose. Nope. They required a brand new prescription from my doctor. I had to beg with my doctor to process it that day. They did not even answer any communication until hours later.”
	Pharmacists do not share information on availability	“In my area, you’ll be hard pressed to find a pharmacist who will give any information about availability unless you already have a prescription electronically transmitted to the pharmacy. Some of those pharmacies are already filling on multi week backlogs. The pharmacists here also will not tell you if they have other strengths in stock, or if other stores in their network have inventory.” “They will not tell you if they have it over the phone, but they told me to have my dr office call to send the prescription over!.” “For the safety of the workers. They will not tell you what they have in stock so they do not get robbed at gun point. You’ll have a better time asking in person. Or forcing your prescriber who is being paid to see you ask for you.”
	Challenges with insurance and prior authorizations	Removing coverage does not always mean insurance will never pay for it. Drop in on r/healthinsurance to verify, but typically it just means you or your doctor need to go to further lengths to demonstrate that this and no other alternative will work for your symptoms, or is tolerable for other reasons such as allergy. I’ve waited 2 weeks for them to get more in and they have not. My insurance also does not cover the name brand, but my HSA will. So this month I had to pay $200 out of my HSA just to be functional.
Trying to understand the causes	Limits on production and restricted access	“While I think the DEA is pointless, the shortage is not simply because they will not increase production allowances. They’re most likely not upping the manufacturing allowances because a few of the several manufacturers of Adderall (both name and generic) still have stock available *on top of* not yet reaching their individual production limit. It’s why the shortage has been so very much location-based so far, because different manufacturers generally supply different regions.” “Wholesalers are restricting access. It explains why manufacturers have not met their quota but no one has enough. Everyone is scared of the DEA and restricting supply in multiple ways.” “This is from the government [website]. They’re saying that the DEA is aware of patient reports of shortages and will work with manufacturers when they have to, but those manufacturing quotas/limits have not been met yet. So this is 100% attributable to manufacturers. They’re not making enough and blaming their failures on guvmint regulations.”
	Impact of COVID-19 supply chain disruption	“There appears to be an issue with getting the raw materials needed to *make* the drugs. No amount of increased labor, money, or advocacy is going to speed up production because *there is simply not enough material to go around*.” “It was caused by a COVID-related labor shortage at Teva, an uptick in demand, and DEA imposed regulations on production. Mostly that last one. The vague misinfo flying around in the press taken from the DEA and the drug companies and the prescribers all blaming each other. I did like a 3 day hyperfocus on it trying to find actual info and now that I have its maddening to see how much people have been misled and are repeating misinfo in all these threads.”
	Increase in overall prescription for ADHD	“The major issue is there was a 20% increase in prescribing the drug without proper psychological testing due to the pandemic restrictions being eased. Telehealth companies like Cerebral and Done pushed nurse practitioners to write prescriptions for stimulants on an average of 30 people a day according to a whistle blower. These same companies are incentivizing influencers to advertise 30 min evaluations on places like Instagram and TikTok.” “.so from my understanding the reason why we have a shortage is due to a telehealth company that did not understand how adderall effects those with adhd, so they planned to increase customer retention by prescribing stimulants to 100% of its adhd patients … … if it makes you feel any better though Kyle Robertson the ex-CEO that Truebe claims “Directed Cerebral Employees find ways to prescribe stimulants to increase retention” is currently being sued for 25 mil.” “So before COVID, there are certain substances called controlled substances, like Xanax and Adderall, that you cannot get through a TeleMed appointment. That’s why I always have to go into my doctor to get my prescription, and you especially cannot get diagnosed through TeleMed for those things. But they lifted that law during COVID cause they had to. And this [expletive] guy Kyle Robertson (CEO of Cerebral) Just started over prescribing everyone Adderall and other drugs for profit.”
	Pharmaceutical industry	“I lost an awesome job of 20 years because of that. Blame the insurance companies and big pharma, not the little guys.” “I wonder if there needs to be more manufacturers or suppliers of ingredients? Or is it like most businesses still recovering from COVID? I’m just skeptical of big pharma who loves to give the power of a drug to one big company and lock it out/make it inaccessible to others.” “The DEA had good intentions to stop addicts, but as always corruption ruins everything. Over 75% of the DEA is funded by Big Pharma, so you can guarantee the board is in their pockets. Probably keeping the amount allowed low on purpose so pharma companies can sell the name brand at an insane markup.”

The interactions with healthcare providers and insurance companies added another layer of complexity to the challenging situation. Some healthcare providers were reported as helpful in assisting patients in navigating these obstacles. Many other redditors reported healthcare providers as unsupportive, leaving individuals to fend for themselves. The necessity to call doctors to change medications or dosages due to the shortage was common. However, it was not always successful. Additionally, insurance companies often required prior authorization for medications or attempted to switch patients to usually unavailable generic alternatives. The financial burden of paying out of pocket for medications, when insurance failed to cover the medication expenses, usually the available brand-name medication, was prohibitively expensive for many.

While scant and conflicting evidence for specific contributors to the ADHD medication exists, redditors speculated on various factors that contributed to the shortage. Redditors discussed strict control over the production and prescription of scheduled drugs, limited manufacturing by big pharmaceutical industries prioritizing profits over supplies, the supply chain impact of COVID, and other market forces as factors affecting the production. Some redditors even implied that the increase in demand of ADHD medication prescription resulting from improved access or misuse of telehealth services leads to the current shortage.

Coping strategies to manage the impact of ADHD medication shortage: in response to medication shortage, redditors described a various strategies to adapt to their situation (Table 3). “Problem-focused coping” included managing access to medications, advocacy, and community support. “Emotion-focused coping” included acceptance, positivity, and social support. Many redditors stated that they would either reduce the dose or skip a dose on non-working days and save the medication for use later when they could not get their medication. Other redditors discussed working with their healthcare providers to change their medication type (to a more available stimulant or non-stimulant), reducing or increasing the dose (to a more available dose regimen), or changing the dosage from immediate-release to extended-release formulations or vice-versa based on the availability. Redditors also shared alternative methods to manage ADHD symptoms, such as the use of caffeine, nicotine, exercise, and meditation. Some even suggested acquiring the medication from dealers or buying it from other countries.

**TABLE 3:** Coping strategies to manage the impact of ADHD medication shortage.

Problem-focused coping		
Managing access to ADHD medication	Switching medication	“People switching to extended release have now caused a shortage in my area.” “I have only been able to get my IR refilled. (And it is very sporadic) It’s been almost 2 months since my pharmacy has gotten ER. It’s not great, but it’s also not as bad as I thought.” “They did say I could call my doctor and ask her to write me a script for IR instead, since only XR is backordered there, but IR does not work super well for me since I always forget the second dose.” “In December, I switched to ER Adderall due to my IR being out. This month can get IR but not ER, and I ended up liking the ER better.”
	Changing dosage	“i typically take vyvanse 70 mg but i could only afford that last year after reaching my insurance deductible, so this year i had my psychiatrist switch to adderall XR 20 mg 2x daily … CVS told me they have not had that in stock for months but said they have plenty of 25 mg XR so that i should have my psych send that in instead. Which she did, i had no problem picking up 60 ^−ΔΔCT^ of 25 mg XR, super weird that it’s only that dose effected so to anyone unable to get their meds filled i recommend asking your prescribing physician to try switching to a higher but very similar dosage! ” “Was supposed to start my first med today, concerta 18 mg. Of course it’s out of stock, but they said they have 27 mg in stock. Hope my Dr will write a script for that dosage and do it quickly.”
	Stopping medications	“Not gonna lie, this shortage is making me happy I had to kick the stuff. The more hurdles between me and that beautiful drug the better.” “Long story short, I stopped taking them cold turkey for about a week, thinking they would restock them. That did not happen so I tried Vyvanse and felt absolutely no different. My doctor increased the dosage, but I hate it. It gives me a terrible headache, and I feel nauseous all day. I was so sick that I quit my job.” “I stopped taking my ADHD medication because of this and HAVE GOTTEN VIRTUALLY NOTHING DONE IN OVER TWO MONTHS. THIS IS MISERABLE.”
	Skipping medication	“I started rationing my Adderall months ago when I first heard about the shortage. What I did was delay taking it in the morning by a couple of hours and skip the afternoon dose unless I really needed it. I’d just load up on caffeine those afternoons. It finally hit here last month and I’m well-prepared.” “I have now stopped taking mine on weekends and holidays in an attempt to ration it for work days. Major sucks as it means I literally sleep the entire weekend and feel kinda sick.” “I tend to ration my meds on the weekends and try to do medication vacations a few days a month. That way, I have enough to hold me over. It’s not perfect, but it’s a strategy.”
	Non-pharmaceutical alternatives	“Time to see my old friends, cigarettes and coffee.” “On breaks I do low stimulation things. Meditation, productive reading, push ups, step outside for a minute, splash cold water on my face, take a 5 min power nap, etc. Plus when you are bored, your mind gets more creative and comes up with new things to do. ” “Caffeine + exercise, which is good for trimming off the jittery edge, if that makes sense. I recommend a briskly paced 1 h walk, maybe while listening to lectures related to your exams/labs/papers?” “That or ‘stock up on Red Bull!’ Like, yeah it helps for attention somewhat, but I’m trying to regulate my dopamine, not get heart palpitations trying to work on my year end performance review. I’m lucky enough to have a tiny booster dose that I rarely need, so reserves of that have lasted this past month and will get me to Jan 5th.”
	Use of illicit supply/obtaining it from other countries	“So … is it time we learn how to find drug dealers or something? Cuz some of us lost jobs or are barely holding onto their jobs.” “A non-dealer/non-abuser person who is in school to become a physician is genuinely considering smuggling medication into the country … because they cannot find a pharmacy to fill the *legal and legitimate* prescription they already have for said medication.” “But think of it this way, why is there street demand in the first place that makes reselling profitable? Why not just allow anyone who wants a script, regardless of diagnosis, to get one cheaply and easily? How many people are buying from those sellers because they have ADHD and cannot afford a doctor, etc.,? And then if some people get high from it who do not have ADHD, I’d rather they do it with a safe pharmaceutical script than something cut and laced by a dealer.”
Advocacy and community support	Petition government	“I’m assuming we need a political campaign and people need to start calling their senators, etc. Not sure if a petition of that sort has already happened. Obviously its been in the news but does not seem like much is happening to remedy it.” “While I am far from the “decriminalize everything” crowd, restrictions need to be seriously re-evaluted. I recall a decade or so ago reading a story about non adhd people ranging from students to CEO’s using these things. The comments that followed had ADHD’ers saying that this is why they have to pay more.” “I agree that there should be some sort of legal action to fight this. Since ADHD is classified as a disability, this probably does not mean the ADA guarantees access to medications. Maybe some sort of public pressure can be put on the DEA and the pharma industry that they have colluded in secret to deny medications to people with disabilities. Or maybe some sort of anti-trust suit stating the pharma industry and the DEA has set artificial limitations to limit competition.” “There are literally 1.7 million members on this subreddit—at the end of the day, I think this is a political issue because pressure is needed on the DEA and FDA. the manufacturers want to sell meds, some have had issues with the actual production and that’s caused issues but ultimately I think it’s because they have quotas that are not in line with reality to say the least. If people on here start making it known through letters, emails, petitions, etc. It will get noticed, I assure you. I humbly suggest starting an association to represent people with legitimate ADD/HD Diagnoses that require medications that are broadly mentioned here and to raise funds to donate to politicians—it will get their attention.”
	Share resources	“For anybody switching to vyvanse and is intimidated by the cost, look up the Takeda help at home program. It’ll bring the cost down to 0. The income threshold is pretty high (40–50k pls do not quote me though) and it’s a form you print and fill out. You attach income statements like your tax returns or something and take it to your doctor. They fill out the rest of the form and fax it in. Discount card comes in about 3 weeks. There is also a 30$–60$ coupon on the vyvanse website. GoodRX will also help bring it down. It took my first script from 434 to 330.” I switched to Vyvanse after I could not find Adderall. So far so good. Thank you for posting this.
	Support community	“I see there is a lot of discussion related to if you should tell your supervisor about having ADHD or not. That truly depends on the situation or person. In this situation, I would first speak with your Psychiatrist to discuss the shortage on medication and need for work accommodations and request them to write a letter stating the need for accommodations at work such as private office/cubicle, quite hours to work, set designated break times, and/or extended time to complete work assignments as needed appropriate or needed. Then you can a draft a formal email to Human Resources department with a request for work accommodations based on the letter from your Psychiatrist. Then meet with your Direct Supervisor to follow up on the needed work accommodations. In that manner you have formal medical documentation of a valid medical disability of Attention Deficit and Hyperactivity Disorder with reasonable work accommodations being provided per the Americans with Disabilities Act by Human Resources, in which you cannot be fired or terminated if you can perform the work required with the reasonable work accommodations per the US Department of Labor rules and laws. Lastly, after that process you have your own personal choice based on how you feel and your professional relationship if you tell your direct supervisor that you have ADHD as it is always better for you to open and honest instead of someone suspecting and ask, but you are legally not required as only Human Resources is needed to be informed of a valid medical disability and need for work accommodations based on the documented disability.”
Emotion-focused coping		
Acceptance and positivity	Understand what it feels like and self-acceptance	“Let’s not forget those “drug abusing degenerates” are self-medicating and are truly not the enemy here, nor is anyone using any amount of drugs. This is a moral panic that denies the existence of real mental health conditions so cops and Karen’s can play armchair doctor to impose their limited world view on the rest of us unfortunate enough not to be in power.” “I lost contact with all college friends/did not text anyone during that period of time (almost a year) when I felt my sickest before getting a diagnosis. It was lots of thinking, which meant a lot of self acceptance, self forgiveness/radical forgiveness, radical acceptance for my circumstances, and becoming a bit protective of myself—as odd as that may sound. I used to (still often fall back into the habit when with siblings and their spouses) of making fun of myself/my “lesser than” qualities for the sake of laughs—something I’m still working on. But accepting learning to love myself for exactly who I am was one of the best silver linings of the hellish years that being so sick COULD have been—had I not been open to any potential positives the experience could bring!” “This does not really relate to OP’s question but I know the internet has 1,000% ruined me because I *really* appreciated your acknowledging not knowing what it’s like in their skin … and particularly in this situation, it’s a real life example of that “treat the janitor (OP) how you would treat the CEO (you)” business lesson. I feel like so many people (guilty of this myself at one time or another, I’m sure) these days are simply just lacking this mindset. It’s truly an endearing quality, personally and professionally! do not ever let that dull!”
Social support and validation	Thankfulness for support	“Please try to remember that your experience is unique to you, and some people may not have the same downsides or negative effects. ADHD medications have liberated and restored life for many. I hope you find healing on your journey off of them, but they are still extremely beneficial to some. <3” “I really feel for you, and I’m sorry you’re going through that. Unfortunately, I do not think feelings are to blame. Medicine is a business in the US. It’s no excuse, but it’s just an inconvenient truth.” “I’m so sorry its been so long … thank you for your positivity! We know the drill. Write lists and aim your arrow. Shoot your shot. Stay off your phone. Find dopamine in rewards after a task is completed. Define those rewards yourself***. Affirmations!! I’m crawling out of paralysis mode so all of the tools are on the table.” “I do not have questions or comments. I just want to thank you for taking the time to do this.”

Many redditors shared information on where and how to locate medications, how to approach pharmacies, recommended using paper scripts, using coupons to reduce medication costs, and shared information about patient assistance programs enabling other ADHD patients to avoid unnecessary hurdles in their search for medications. In addition, redditors petitioned lawmakers to improve production and access to ADHD medications.

In response to the challenges posed by the ADHD medication shortage, community support has played a vital role in aiding and supporting the affected individuals. Several posts from redditors showed support for those who were experiencing the shortage. Support included words of positivity, optimism, guidance, and emotional support to those affected. Many redditors used the social network to share their feelings and experiences, receiving and providing supportive feedback that fostered catharsis and a sense of solidarity.

## Discussion

Amid an unprecedented medication shortage, findings from our study of Reddit posts show that individuals with ADHD faced severe consequences resulting from ADHD medication shortage. When consumers encounter shortages, their reaction is primarily to reduce the consumption and increase the storage or purchase of a specific good. With ADHD medications, both options are challenging to perform. Any reduction in medication use leads to a decline in cognitive ability, and additional action to obtain ADHD medication becomes almost impossible due to the strict regulations imposed on the prescription and fills of such medication. Therefore, the shortage left ADHD patients with limited options and significantly impacted their quality of life.

Our findings illustrate how ADHD medication can be understood through the lens of the stress and coping theory. In the primary appraisal phase, redditors consistently evaluated the shortage as being harmful and disruptive to their quality of life. They described severe emotional and cognitive impacts, leading to anxiety, depression, and an inability to function, which is a hallmark of the primary appraisal. The narratives underscore the serious threat that the lack of medication pushes patients into an inescapable cycle of misery. In the secondary appraisal phase, redditors assessed available resources and their capacity to respond to the shortage. Many redditors experienced significant structural barriers, medical administrative hurdles with providers, pharmacists, and insurance providers, and a lack of support and stigma from healthcare providers and family and friends alike. Additionally, activities that were under self-control such as executive functioning to maintain medication fills were impacted due to medication shortage. In response, redditors employed a range of coping strategies such as reducing the dose, changing the medication, skipping the dosage, and using alternative ways to suppress their symptoms. In addition, the Reddit community has come together to suggest collective actions to improve the production and access to ADHD medication, sharing resources to navigate bureaucracy and providing support and validation to other individuals impacted by the medication shortage.

Beyond the stress and coping theory, these findings align with broader frameworks in public health research, including theories related to the sociopolitical regulation of controlled substances, which, while intended to protect patients, are often unhelpful and in many cases cause additional harm (Dickson-Gomez et al., 2022; Kanouse and Compton, 2015). These models help contextualize the role of stigma, systemic barriers, and policy-level constraints in shaping individual experiences and responses to medication shortages. The analysis of Reddit posts reveals a clear distinction between adaptive and maladaptive coping strategies to manage ADHD medication shortage. The adaptive strategies included planned use of medication to have surplus, community support, and resource navigation, which reinforce planned behavior, forming social bonds, and empowering individuals facing systemic challenges. In contrast, the maladaptive strategies included using alternative medications without medical guidance, discontinuing treatment completely, using caffeine and tobacco, and social withdrawal, reflecting avoidant strategies, which may ultimately lead to poor mental health outcomes and in severe cases such as purchase of illicit medication—death. The interplay between the limited options and urgency of managing symptoms of unmedicated ADHD led many redditors to adopt coping strategies, which, while understandable, exposed them to further harm. Such maladaptive behaviors have been noted in other resource-constrained environments such as insulin underutilization (Herkert et al., 2019), delayed healthcare utilization (Ratnapradipa et al., 2023) due to cost, underutilization of medication for opioid use disorder due to stigma (Madden, 2019), or pregnancy termination without medical supervision due to restrictive abortion laws (Harris and Grossman, 2020).

While research on this area is scant, a recent qualitative study published by Johnson et al. including 20 participants. highlighted a similar set of challenges, such as the impact on life that includes ADHD symptoms, impact on productivity, emotional effects, and challenges with social relationships and activity (Johnson et al., 2024). The study also highlights treatment barriers (stigma from providers and lack of information), challenges with accessing medication (switching medications and traveling long distances), and coping strategies (exercise and non-medical routes/illicit medications and meditations). The findings from this study support our findings and highlight the immense consequences of the medication shortage.

A recently published report on MMWR highlighted that in 2023, 6.0% of US adults received a diagnosis of current ADHD (Staley et al., 2024). Approximately 50% were prescribed medications in the last year for their condition, and among those individuals, 71.5% had challenges in filling their prescriptions, highlighting the extent of ADHD medication shortage. The study also highlights that almost 46% of the individuals reported telehealth for ADHD care (Staley et al., 2024). During the pandemic, the DEA and the US Department of Health and Human Services allowed for flexibility in stimulant prescribing via telehealth, which could have resulted in an increase in ADHD diagnosis and management. However, some online telehealth companies were able to use the relaxed guidelines to provide stimulants to patients who did not need the medications. The Justice Department is leading a case against such companies, providing some credence to the speculation of redditors (Office of Public Affairs, 2024). Since the filing of these charges, the CDC has warned of disruption in services leading to “injury and overdose” (CDC Health Alert Network, 2024). The CDC warns that there is an increased risk of overdose due to contamination of fentanyl, with new reports suggesting that seven in 10 pills seized by the DEA contain fentanyl (Drug Enforcement Administration, 2022). Since most ADHD patients who seek illicit medication may not have knowledge of the contamination and do not have access to naloxone, any unwanted exposure may lead to a fatal overdose. Therefore, it is essential that ADHD patients who are facing medication shortages are warned of the risks.

A systematic review published in 2019 highlighted the economic impacts on patients, institutional cost increases, adverse clinical outcomes, and adverse events due to medication shortage (Phuong et al., 2019). Although none of the studies included in the review directly focused on ADHD medication shortage, major findings highlighted in the review, such as increased out-of-pocket cost due to brand switching or alternative medicine or increased travel to obtain medication and adverse “humanistic outcomes” such as anxiety and frustration, were commonly reported in people experiencing medication shortages (Phuong et al., 2019).

The current medical system, which restricts access to necessary medication by adding unnecessarily complex approval processes that involve physicians, pharmacists, and insurance companies, places an undue burden on patients, particularly during medicine shortages. With the lack of medication, adversely affecting the patient’s ability to actively search for and obtain approval to receive the medication, many ADHD patients cannot get the treatment they need. It is, therefore, necessary to introduce a streamlined treatment system while maintaining adequate supervision from healthcare providers. Strengthening the connection between medication demand and supply through mandatory alerts and transparency between pharmaceutical companies and regulatory bodies can help prevent shortages. Additionally, expanding insurance coverage for non-pharmacological treatments, such as behavioral therapy and cognitive training, offers alternative symptom management strategies during medication shortages. These policy changes can provide critical insights to make informed decisions and improve health outcomes.

The use of Reddit posts for the evaluation of ADHD medication shortage serves not only as a timely case study but also as a powerful example of how social media can function as a valuable tool for public health surveillance and insight generation. Through user-generated content, platforms such as Reddit and Twitter can offer real-time and often unfiltered accounts of individuals’ perceptions, experiences, struggles, thought processes, and coping strategies. These “digital narratives” provide contextual information that may not be captured through clinical records or surveys. Therefore, these posts can be vital in forming and shaping policy responses, healthcare provider awareness, and future research on medication access, use, and adherence. Broadly, these findings highlight the relevance of social media as a complementary source of understanding and tackling emerging public health challenges.

## Limitations

While our study performed an extensive review of Reddit posts to examine the impact of ADHD medication shortage, one of the main limitations of our study is that all the posts are considered “self-report” and could be biased. We cannot confirm the veracity of the statements in the Reddit posts. In addition, the findings reported in our study exclude comments that refer to the shortage or availability of ADHD medication outside the United States. Furthermore, we cannot quantify the overall period of shortage experienced by the redditors who posted about the shortage and when the supply challenges are resolved. Future studies should examine ADHD medication prescription fills data to examine the impact of the shortages. Additionally, the stress and coping theory, as applied in this manuscript, only focuses on a few key themes per post rather than a continuum of appraisal and coping strategies in each individual.

## Conclusion

ADHD patients experienced significant health impacts due to the ADHD medication shortage. The experiences reported by redditors allowed us to get a unique perspective on how the medication shortage impacted individuals with ADHD. Our research findings highlight that proper measures to ensure continued access to critical medications are necessary among ADHD patients. Furthermore, when a widespread shortage of medications occurs, rapid public health intervention is needed to inform patients, support treatment, and reduce barriers to access.

## Data Availability

The data used in this study are publicly available on Reddit. Further inquiries on the specific instance of the data used can be directed to the corresponding author.
